# Clinical outcomes of patients with Garden I and II femoral neck fractures as verified on MRI: a retrospective case series

**DOI:** 10.1186/s12891-022-05088-0

**Published:** 2022-02-12

**Authors:** Jonas Sundkvist, Pontus Sjöholm, Ana Pejic, Olof Wolf, Olof Sköldenberg, Cecilia Rogmark, Sebastian Mukka

**Affiliations:** 1 Department of Surgical and Perioperative Sciences, Orthopedics, Umeå University, Umeå, Sweden; 2 Department of Orthopaedics, Lund University, Skåne University Hospital, Malmö, Sweden; 3 Department of Surgical Sciences, Orthopaedics, Uppsala University, Uppsala, Sweden; 4 Karolinska Institutet, Department of Clinical Sciences at Danderyd Hospital, Unit of Orthopaedics, Stockholm, Sweden

## Abstract

**Background:**

Between 2 to 10% of non-displaced femoral neck fractures (nFNF) cannot be diagnosed on plain radiographs and require further imaging investigation to be detected or verified. These fractures are referred to as occult hip fractures. This study aimed to report treatment failures, reoperations and mortality in a consecutive series of occult femoral neck fractures (FNF) treated with internal fixation (IF).

**Methods:**

A retrospective multicenter study was performed based on a consecutive series of patients aged ≥ 60 years with an occult magnetic resonance imaging (MRI) verified Garden I and II FNF sustained after a trauma and treated with primary IF. We included 93 patients with a minimum 2-year follow-up. Radiographic assessment encompassed pre- and postoperative tilt, implant inclination, MRI and treatment failure. Data on reoperation and mortality were collected. Treatment failure was defined as fixation failure, nonunion, avascular necrosis or posttraumatic osteoarthritis.

**Results:**

The study comprised of 93 patients (72% women, 67/93) with a mean age of 82 (range, 60–97) years. Overall, 6 (6%) patients had major reoperations. 2 (2%) had minor reoperations. One-month mortality was 7%, 1-year mortality was 20% and 2-year mortality was 31%.

**Conclusion:**

This multicenter cohort study identifies a subgroup of elderly patients with MRI verified Garden I and II FNFs sustained after trauma, i.e. occult fractures. These fractures seem to have a lower complication rate compared to nFNF identified on plain radiographs.

**Level of evidence:**

Prognostic Level V. See Instructions to Authors for a complete description of levels of evidence.

## Background

Femoral neck fractures (FNFs) are commonly encountered in orthopedic practice and the absolute numbers are expected to increase further as there is a growing elderly population worldwide [1]. Most FNFs can receive adequate treatment after being diagnosed with plain radiographs [2]. A small group of non-displaced (nFNF) or minimally displaced FNFs sustained after trauma cannot be diagnosed on plain radiographs and require further investigation with computed tomography (CT), radionuclide bone scan or magnetic resonance imaging (MRI). These fractures are referred to as occult hip fractures and represent 2–10% of all nFNF [2–6]. MRI is more accurate than both CT and radionuclide bone scans to detect occult fractures and also reduce time to diagnosis [2, 7, 8]. Reoperation rates after internal fixation (IF) of nFNFs detected on plain radiographs range from 8 to 19% in previous reports [9]. However, there may be subgroups of nFNFs whose fracture characteristics may lead to different outcomes, complications and reoperation rates [10, 11]. The MRI verified nFNFs, i.e. the occult hip fractures, is a potential subgroup. There are few reports on the outcome of these fractures [12, 13]. Therefore, our study aimed to describe treatment failures and reoperations in patients with MRI verified FNFs treated with IF.

## Method

### Study settings

A retrospective multicenter cohort study was performed including patients ≥ 60 years with an MRI verified FNF treated with IF between January 2003 and October 2018 at four orthopedic departments in Sweden: Umeå University Hospital (2003–2018) a third-level university hospital with a catchment area of about 160,000 inhabitants, Danderyd Hospital (2010–2018) a third-level university hospital with a catchment area of about 500,000 inhabitants, Skåne University Hospital in Malmö (2005–2014) a third-level university hospital with a catchment area of about 450,000 inhabitants and Skellefteå Hospital (2004–2018) a first-level hospital with a catchment area of about 80,000 inhabitants.

### Patients and data collection

A consecutive series of patients ≥ 60 years with an MRI verified FNF were included. Only patients treated with IF by either cannulated screws or pins were included and followed until death or December 2020. Patient demographics were collected by a review of the surgical and medical charts. We collected data including age, sex, ASA classification, cognitive impairment (diagnosis in medical records prior to fracture), use of a walking aid prior to fracture, admission from sheltered housing or a nursing home, the use of MRI for diagnosis, method of surgical treatment, reoperation, treatment failure and date of death.

### Radiographic assessment

**Fig. 1:**
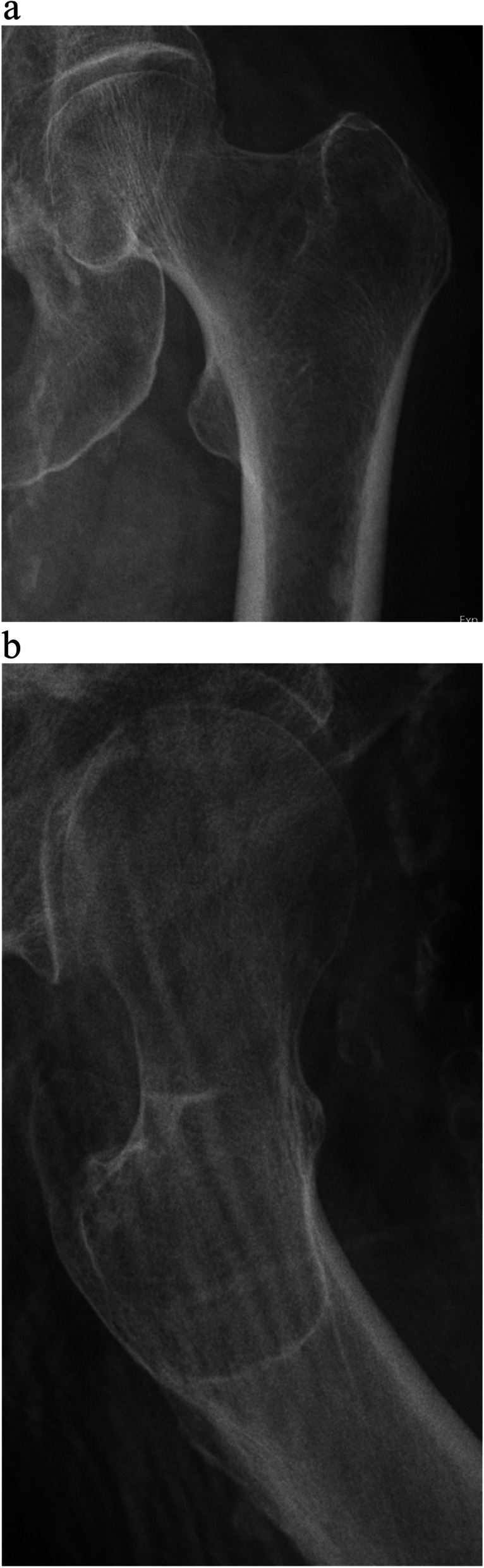
Plain radiographs of an occult femoral neck fracture. **a**) AP projection of the hip. **b**) lateral projection of the hip

**Fig. 2:**
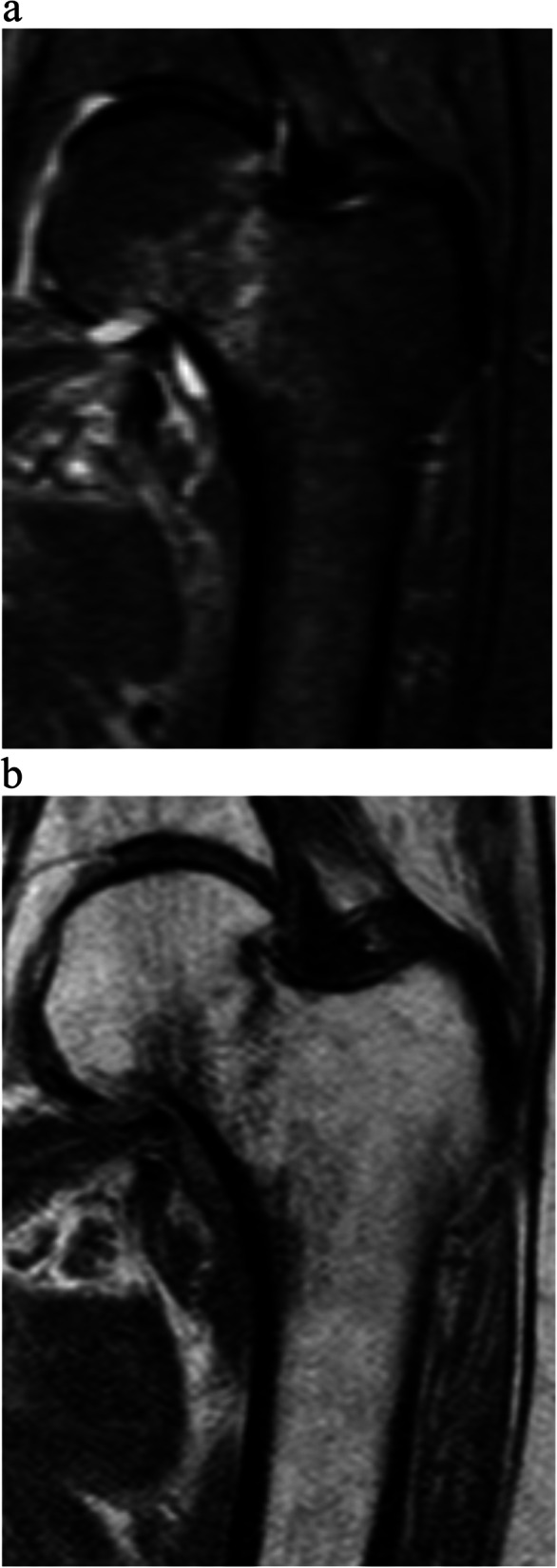
MRI of the same occult femoral neck fracture. **a**) T2 weighted anteroposterior image. **b**) T1 weighted anteroposterior image

The plain anteroposterior (AP) radiographs were used to classify fractures according to the Garden classification system (Fig. 1 a, b) [14]. The pre- and postoperative tilt of the femoral head was measured on a lateral radiograph of the hip using the method described by Palm et al. [10, 11, 15]. If a postoperative lateral radiograph was missing, the postoperative tilt was measured on the intraoperative image documentation. For implant inclination we performed measurements on the inferior pin or screw on the postoperative AP radiograph [16]. Three raters (JS, PS, AP), who were not blinded, performed all measurements. At the time of the study no national guidelines on diagnosing fractures with MRI were present, however, in most cases T1, T2 and STIR weighted sequences were used (Fig. 2a, b). All images were digitally acquired using a Picture Archiving and Communication System (PACS, Impax, Agfa, Antwerp, Belgium).

### Internal fixation

IF was performed according to the same principles at the 4 hospitals. With the patient on a fracture table and under intra operative imaging 2 or 3 pins/screws were placed along the femoral neck transfixing the fracture. Either Hansson Pins; Swemac Orthopaedics AB, Sweden or Olmed Screws; DePuy/Johnson & Johnson, Sollentuna, Sweden were used.

### Outcome measurements

The primary end-point was a major reoperation due to avascular necrosis (AVN), fixation failure, posttraumatic osteoarthritis or nonunion. Major reoperation was defined as hip arthroplasty, excision arthroplasty or re-osteosynthesis due to peri-implant fractures. The definition of minor reoperation was removal or adjustment of implant.

### Statistical analysis

Variables are presented as proportions of all fractures. Nominal variables are presented as proportions of all fractures and scale variables as means ± standard deviation (± SD) and range. We used SPSS (IBM SPSS Statistics for Mac, Version 26.0, Armonk, NY: IBM Corp. USA) for statistical analyses.

## Results

### Patients and descriptive data

We included 93 patients (72% females) with a mean age of 82 (range, 60–97) (Table 1). The median follow-up was 74 (range, 0–190) months. One-third of the patients suffered from cognitive impairment and 23% were admitted from sheltered housing. The 30-day mortality was 7%, 1-year mortality was 20% and 2-year mortality was 31%.

**Table 1:** Patient characteristics: Distribution of sex, age at injury, cognitive impairment and sheltered housing

Patient characteristics (*n* = 93): Data are presented as median and range or the number of the patients with percent in parentheses	
Age	84(60–97)
Women	67(72%)
ASA classification	
1–2	26(28%)
3–5	63(68%)
Missing	4(4%)
Cognitive impairment	31(33%)
Sheltered housing	21(23%)

ASA = American Society of Anesthesiologist

### Radiographic assessment

We found all of the included fractures to be non-displaced on the AP radiograph and used the method described by Palm et al. [15] to verify that the fractures were non-displaced or minimally displaced on the lateral radiograph (Table 2).

**Table 2:** Fracture characteristics: Degree of dorsal tilt preoperative and postoperative and implant inclination

Patient characteristics (*n* = 93): Values are given as median and interquartile range	
Preoperative tilt	4°(7)
Preoperative tilt	3°(7)
Implant Inclination	135°(11)
≤ 125°^†^	7(8%)
> 125°^†^	86(92%)

^†^The values represent the number of the patients and percent in the parentheses

### Treatment failure and reoperations

**Fig. 3:**
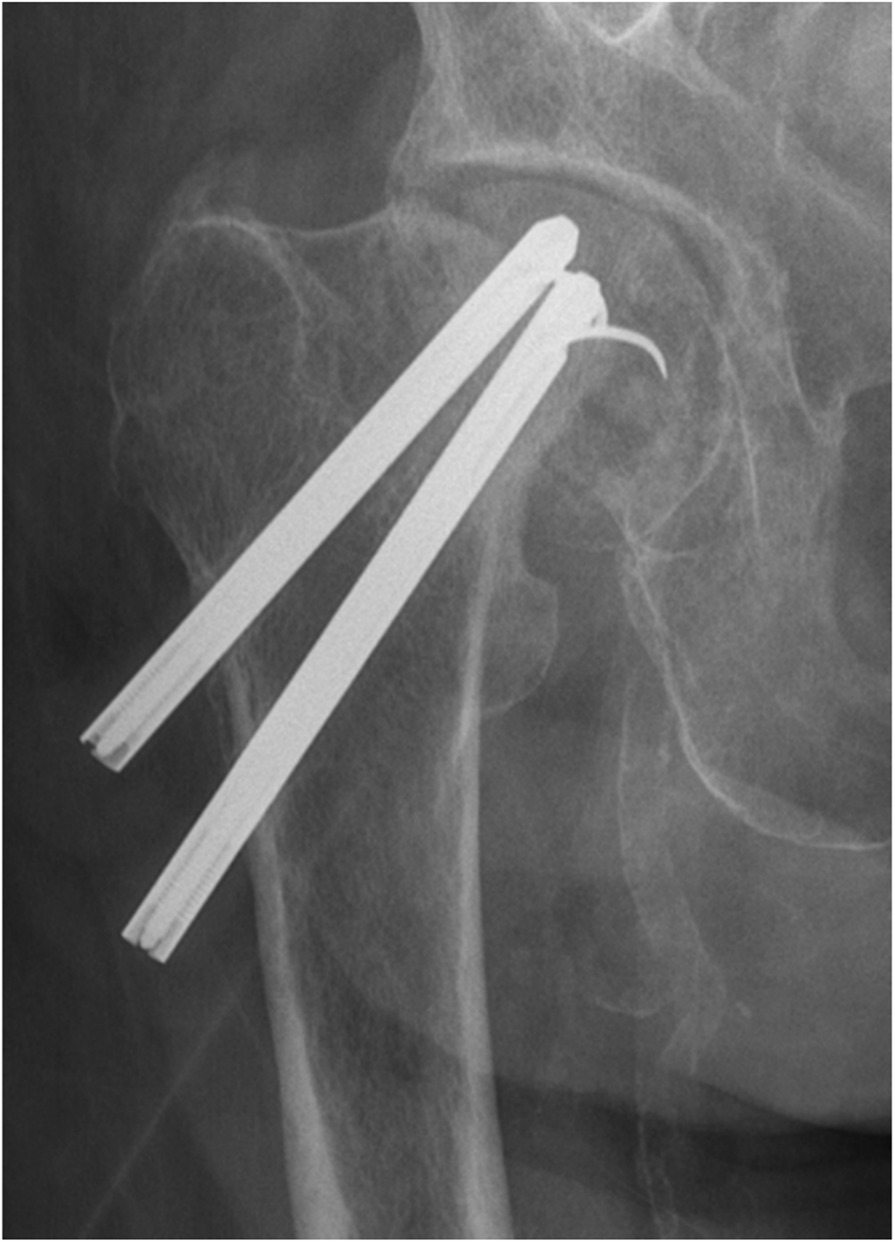
Plain radiographs of an occult femoral neck fracture treated with internal fixation and who later developed a treatment failure

Overall, 6 (6%) patients were classified as treatment failure and were treated with reoperations (Fig. 3). In total, 8 (8%) patients had reoperation as two patients had implant removal (Table 3). 1 patient with AVN, 1 with non-union, 2 suffered fixation failure and 1 a peri-implant fracture. 1 developed post-traumatic osteoarthritis. 8 (8%) patients underwent a reoperation, including minor procedures during the study period. 6 patients had a major reoperation during the first 2 years after IF, 2 patients received a total hip arthroplasty, 2 patients hemiarthroplasty and 2 patients underwent re-osteosynthesis. Minor reoperation with removal of implants was performed in 2 patients.

**Table 3:** Treatment failure and reoperations

Treatment failure and reoperation (*n* = 8): Values are given as median and range or the number of the patients with percent in parentheses	
Age	83(60–92)
Female	5(63%)
Treatment failure	6(6%)
Avascular necrosis (AVN)	1
Non-union	1
Fixation failure	2
Post-traumatic arthritis	1
Peri-implant fracture	1
Reoperation	8(9%)
Total arthroplasty	2
Removal of osteosynthesis	2
Hemiarthoplasty	2
Re-osteosynthesis	2

## Discussion

The main finding in this study is that MRI verified nFNFs, i.e. occult hip fractures, have a low but not insignificant rate of complications and reoperations when compared to nFNFs verified with plain radiographs [9]. In the present study the complication rate was 6%, major reoperations were evenly distributed between AVN, fixation failure, peri-implant fractures and post-traumatic arthritis resulting in 2 cases of re-osteosynthesis and 4 cases of hip arthroplasty. 2 patients had minor reoperation defined as implant removal. It has previously been reported that nFNFs treated with IF have reoperation rates between 8 and 19% [9]. These reoperation rates are higher than those presented in our cohort due to our selection of patients. We suggest that the MRI verified occult fracture may be a subgroup of nFNF with a relatively low rate of reoperations following IF. Other subgroups of nFNFs treated with IF have been identified with significant reoperation rates. A preoperative posterior tilt over 20° on the lateral radiograph has been shown to increase the risk of treatment failure and major reoperation [17–20]. An anterior tilt of at least 10° is associated with up to 40% suffering a treatment failure [10, 11]. However, these reoperation rates in combination with the limited literature comparing IF with hip arthroplasty in the elderly population, warrant further comparative studies [21, 22]. Limitations of the present study include the retrospective design. In addition, the limited sample size which prevents us from performing any in-depth analysis of risk factors associated with treatment failure and reoperations. We did not perform any intra- or interobserver reliability testing of the obtained measurements. However, interobserver reliability has been presented in a previous study from our institution [11]. Nevertheless, this is, to our knowledge, the largest consecutive series of MRI verified nFNFs presented to date. The sample size included offers a rough model of the outcome of these fractures which represents the”best possible” clinical results of a FNF treated with IF. We believe our data is highly reliable, as we used the unique Swedish personal identity number to collect data by reviewing the hospital records of all contributing departments. In addition, the hospitals provide all acute orthopaedic care in the catchment area to ensure completeness of data. However, this fragile group of patients, with a relatively large share living in sheltered housing, are often unfit to seek healthcare services actively. This could mask the identification of failures and potential major reoperations related to nFNF.

## Conclusion

Based on our results, MRI verified nFNFs have a lower reoperation rate than nFNF seen on conventional radiographs. Still, MRI verified nFNF are not exempt from hip related complications and clinicians and patients need to be aware that even these perceived benign fractures are at risk of reoperation.

## Data Availability

Due to Swedish legislation, the datasets used and/or analyzed during the current study are not publicly available. Data is available from the corresponding author on reasonable request.

## Abbreviations

NFNF: Non-displaced femoral neck fracture
FNF: Femoral neck fracture
IF: Internal fixation
MRI: Magnetic resonance imaging
CT: Computed tomography
RCT: Randomized clinical trial
ASA: American Society of Anesthesiologists
AP: Anteroposterior
PACS: Picture Archiving and Communication System
SPSS: Statistical Package for the Social Sciences
